# Enhancing the biocompatibility of rhodamine fluorescent probes by a neighbouring group effect†
†Electronic supplementary information (ESI) available. See DOI: 10.1039/d0sc02154g


**DOI:** 10.1039/d0sc02154g

**Published:** 2020-06-22

**Authors:** Jonas Bucevičius, Georgij Kostiuk, Rūta Gerasimaitė, Tanja Gilat, Gražvydas Lukinavičius

**Affiliations:** a Chromatin Labeling and Imaging Group , Department of NanoBiophotonics , Max Planck Institute for Biophysical Chemistry , Am Fassberg 11 , 37077 Göttingen , Germany . Email: grazvydas.lukinavicius@mpibpc.mpg.de; b Department of NanoBiophotonics , Max Planck Institute for Biophysical Chemistry , Am Fassberg 11 , 37077 Göttingen , Germany

## Abstract

Fluorescence microscopy is an essential tool for understanding dynamic processes in living cells and organisms. However, many fluorescent probes for labelling cellular structures suffer from unspecific interactions and low cell permeability. Herein, we demonstrate that the neighbouring group effect which results from positioning an amide group next to a carboxyl group in the benzene ring of rhodamines dramatically increases cell permeability of the rhodamine-based probes through stabilizing a fluorophore in a hydrophobic spirolactone state. Based on this principle, we create probes targeting tubulin, actin and DNA. Their superb staining intensity, tuned toxicity and specificity allows long-term 3D confocal and STED nanoscopy with sub-30 nm resolution. Due to their unrestricted cell permeability and efficient accumulation on the target, the new probes produce high contrast images at low nanomolar concentrations. Superior performance is exemplified by resolving the real microtubule diameter of 23 nm and selective staining of the centrosome inside living cells for the first time.

## Abstract 2

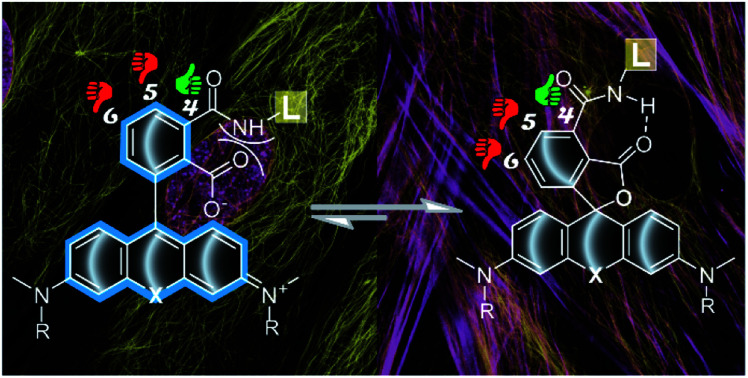
Excellent live-cell staining and nanoscopy imaging with rhodamine 4′-isomer probes boosted by a neighboring group effect.

## Introduction

Modern super-resolution fluorescence microscopy and nanoscopy techniques permit observation of biological processes in living organisms down to the molecular level.^1^ However, their success depends on the availability of biocompatible fluorescent probes for specific labelling of cellular structures. Usually, these probes are constructed by coupling a fluorescent dye *via* a linker to a small molecule ligand targeting a protein of interest. The linker and the fluorophore make significant contributions to the final properties of such probes, often resulting in compromised cell permeability and off-targeting.^2^ Recent studies dedicate a lot of attention to the rhodamine class fluorophores that can cycle between a non-fluorescent spirolactone and a fluorescent zwitterion form. This property is exploited to generate fluorogenic probes, which are sensitive to a number of environmental factors: pH, ion concentration, enzymatic activity, local microenvironment polarity or light.^3^ Since the spirolactone is a more hydrophobic molecule compared to the zwitterion, it makes rhodamine-based fluorescent probes more cell-permeable.^4^ Regular rhodamines and carbon-substituted analogues (carbopyronines) have the equilibrium shifted towards the zwitterion form, which results in relatively poor membrane permeability.^4^ To overcome this issue, several studies attempted to induce spirolactone form preference by introducing electron-withdrawing groups into the xanthene core^5^ or in the benzoic acid substituent.^6^ However, these approaches result in a bulkier core structure and alter the physicochemical properties of the dyes. In addition, all of them must rely on the fluorogenicity in order to minimize background staining arising from the high working concentrations of the probes. We propose a way for modulating the spirolactone–zwitterion equilibrium without any change in the fluorescent dye core structure by exploiting the neighbouring group effect (NGE), *i.e.* the phenomenon that two neighbouring carboxylic groups can influence each other *via* steric, electrostatic or H-bond interactions.^7^ The NGE should not be confused with the term “neighbouring group participation” (NGP) – the direct interaction with a reactive center during a chemical reaction.^8^

Here, we introduce a new positional isomer with NGE to increase the cell permeability and fluorogenicity of regular rhodamines without changing their spectroscopic properties. We demonstrate that excellent quality of wash-free multi-colour live-cell images can be obtained at low working concentrations with wide-field, confocal and super-resolution microscopes even with moderately fluorogenic probes. Selective centrosome staining is demonstrated using exceptionally well performing tubulin probes. Superior performance is exemplified by resolving the real microtubule diameter of 23 nm in STED nanoscopy images.

## Results and discussion

### Chemical synthesis of 4′-carboxyl rhodamines

For a long time, the existence of a novel isomeric class of 4′-carboxyrhodamines was debated due to the synthesis challenge arising from steric hindrance and the ortho-effect altered reactivity of adjacent carbonyl groups.^9^ Isomeric mixtures of 5′/6′-carboxyrhodamines and fluoresceins are usually obtained by the classical condensation process of phthalic anhydrides and 3-dimethylaminophenol or resorcinol respectively.^10^

**Fig. 1:**
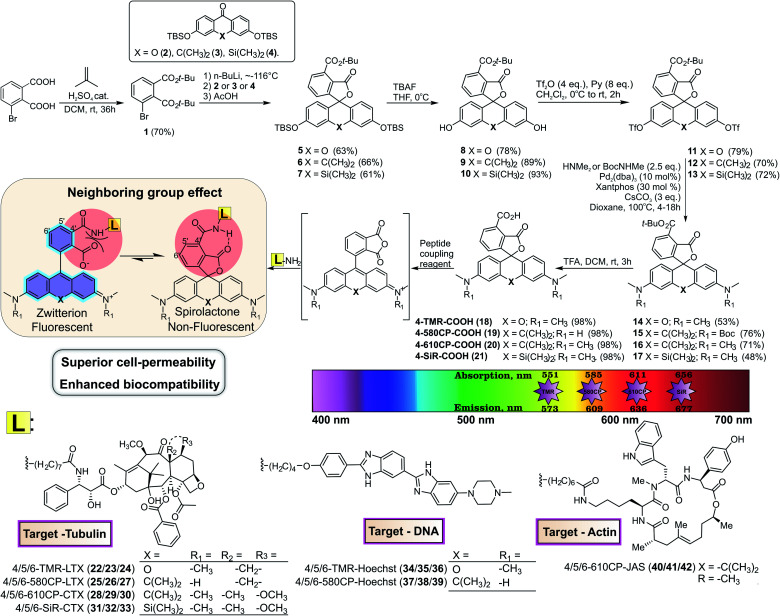
Synthesis scheme of **4-TMR-COOH**, **4-580CP-COOH**, **4-610CP-COOH** and **4-SiR-COOH** dyes and conjugates **22–42**. Their photophysical properties are depicted in the colour spectrum chart and the neighbouring group effect is highlighted.

The condensation between resorcinol and 1,2,3-benzenetricarboxylic acid or its homologs yields solely 7′-carboxyfluorescein and not 4′-carboxyfluorescein.^11^ Another widely applied and regioselective synthesis strategy, which can be applied in the synthesis of 4′-carboxyrhodamines, involves addition of aryl-lithium species to the 3,6-substituted-xanthone.^12^ We used formation of di-*tert*-butyl ester **1** for the protection of 3-bromophthalic acid carboxyl groups for the halogen–lithium exchange reaction (Fig. 1). However, the *tert*-butyl protecting group has low tolerance to strong nucleophiles and the lithium–halogen exchange must be carried out at temperatures between –90 and –120 °C. Such temperatures are not compatible with the reactivity of xanthones and their C/Si analogues possessing strong electron donating dialkyl amino groups at positions 3 and 6. When the halogen exchange was attempted at –78 °C, mainly homo-addition products were formed. To solve this issue, we performed the reaction at ∼–116 °C with the more reactive silyl protected 3,6-dihydroxyxanthone **2** and its C (**3**) and Si (**4**) analogues and performed the late stage fluorescein to rhodamine conversion strategy.^13^ To achieve this, TBDMS protecting groups on compounds **5–7** were removed with TBAF in THF and the obtained fluorescein class compounds **8–10** were reacted with triflic anhydride in the presence of pyridine in DCM to obtain the triflates **11–13**. The triflate groups were converted to the desired (di)methylamino groups (compounds **14–16**) by a Pd-catalyzed C–N cross-coupling reaction and the desired 4′-carboxyrhodamines were obtained by quantitative deprotection with a 1 : 1 mixture of DCM/TFA (Fig. 1). The 4′-carboxyrhodamines behave differently than other regioisomers upon activation with common peptide coupling reagents – the formed carbonyl activated species (NHS ester, HOBt ester or similar) immediately undergoes intramolecular cyclisation forming the anhydride (Fig. 1 and ESI Fig. 1†). In the presence of amines as nucleophiles the major products form *via* nucleophilic attack on a less hindered carbonyl group (position 4′), although traces of spiroamide products can be observed by LC/MS analysis.

### *In vitro* characterization of the neighbouring group effect in 4′-carboxyrhodamines

4′-, 5′- and 6′-Carboxyrhodamine regioisomers have almost identical absorption, fluorescence emission spectra and quantum yield (QY) in phosphate buffered saline (PBS) (ESI Table 1†). In agreement with previously published observations, we obtained an excellent correlation between QY and fluorescence lifetime (ESI Fig. 2a†).^14^ Even better correlation was obtained between fluorophore brightness (product of QY and extinction coefficient) and fluorescence lifetime with the exception of the **4-SiR-COOH** dye, which showed a significantly lower extinction coefficient (ESI Fig. 2b†).

To characterize the ability of the fluorophores to switch between spirolactone and zwitterion states, we measured the absorbance in water–1,4-dioxane mixtures with known dielectric constants.^15^ We fitted the absorbance change to the dose–response equation and obtained the ^dye^*D*_50_ value, which indicates the dielectric constant at which absorbance is halved (ESI Table 2 and Fig. 3†). ^dye^*D*_50_ of **4-SiR-COOH** was significantly higher than the ^dye^*D*_50_ of 5′- and 6′-isomers. For all other fluorophores, the ^dye^*D*_50_ of all three isomers were very close.

We have previously demonstrated that the performance of the tubulin probes depends on the chosen taxane ligand, and that the optimal ligand is different for each fluorophore.4*a* Based on this knowledge, we synthesized a new series of tubulin probes by coupling the 4′-, 5′- and 6′-regioisomers of **TMR-COOH** and **580CP-COOH** fluorophores to a Larotaxel derivative (**LTX-C8-NH_2_** (**SI-4**)) and **610CP-COOH** and **SiR-COOH** to a Cabazitaxel derivative (**CTX-C8-NH_2_** (**SI-3**)) (Fig. 1). 1,4-Dioxane–water titration of **TMR-LTX** probes yielded bell-shaped dose–response curves, indicating two transitions corresponding to zwitterion formation followed by aggregation (ESI Fig. 4†). These processes are described by ^probe^*D*_50_ and ^probe^*A*_50_ parameters, respectively (ESI Table 3†). If the same titration is performed keeping the SDS concentration constant at 0.3%, the aggregates cannot form and the data can be fitted to a simple EC_50_ equation (ESI Fig. 4 and Table 3†).

**Fig. 2:**
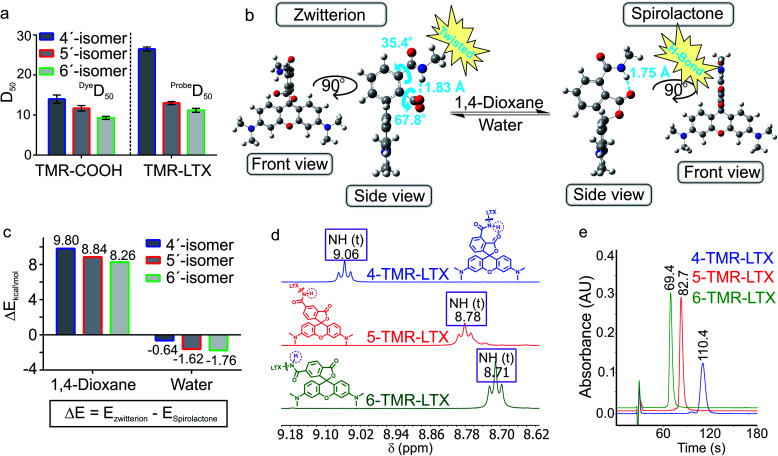
Neighbouring group effect in the fluorescent probes. (a) ^Dye^*D*_50_ values of positional isomers of **TMR-COOH** and ^probe^*D*_50_ of **TMR-LTX**. (b) DFT optimized geometries of a model 4′-regioisomer fluorescent probe in spirolactone and zwitterion forms with a truncated linker and targeting moiety. (c) DFT calculated potential energy differences between the spirolactone and zwitterion of model 4′/5′/6′-regioisomer probes in 1,4-dioxane and water environment. (d) Chemical shifts of the amide proton of **TMR-LTX** regioisomeric probes. (e) Comparison of the retention times of **TMR-LTX** regioisomeric probes in HPLC analysis with an SB-C18 column under isocratic elution conditions (75 : 25 MeOH : H_2_O, 25 mM HCOONH_4_, pH = 3.6).

For 5′- and 6′-regioisomers ^probe^*D*_50_ were only marginally higher than the ^dye^*D*_50_ of corresponding fluorophores. ^probe^*D*_50_ of 4′-regioisomers was significantly increased compared to the corresponding ^dye^*D*_50_ (ESI Tables 2 and 3†). This indicates that the conversion of the carboxyl group to amide induces higher spirolactone content at equilibrium and thus implies higher hydrophobicity of 4′-probes (Fig. 2a).

In order to better understand how NGE alters the spirolactone–zwitterion equilibrium, we modelled *in silico* 4′/5′/6′-carboxamidetetramethylrhodamine methylamides (Fig. 2b and ESI Fig. 5†) with a truncated ligand and linker. Geometrical structure optimizations pointed out unique properties of the 4′-isomer: the spirolactone form can form an intramolecular H-bond since the carboxamide proton and carbonyl are only 1.75 Å apart; the carboxylate is 68° twisted with respect to the benzene ring because of steric hindrance in the zwitterion form (Fig. 2b and ESI Fig. 5†). Comparison of calculated total potential energies of the molecules in the modelled solvent environment representing two uttermost points in 1,4-dioxane (*ε*_r_ = 2.21) and water (*ε*_r_ = 78.35) indicates that the spirolactone form of **4-TMR-NHMe** in 1,4-dioxane is stabilized and the zwitterion form in water is destabilized in comparison with other regioisomers (Fig. 2c). As the stabilization of the spirolactone form could be attributed to the formation of intramolecular hydrogen bonds, the stabilizing effect could be considered diminishing upon increase of water content. Therefore the observed shift of *D*_50_ value in 4′-carboxamide isomers mostly arises from the destabilization of the zwitterion form in the water environment (ESI Table 4†). NMR spectroscopy confirmed the presence of the NGE: the amide proton in the 4′-isomer probes is deshielded and downfield shifted by 0.3–0.4 ppm compared to 5′- and 6′-regioisomers. This shift does not depend on the xanthene ring system or on the attached targeting ligand (Fig. 2d and ESI Table 5†).

The higher ^probe^*D*_50_ values of 4′-regioisomers suggest a higher percentage of the hydrophobic spirolactone form under equilibrium conditions which is retained longer in the reverse phase C_18_ HPLC column because of the stronger interaction with the resin.^16^ We compared retention times of the probes under isocratic elution conditions and observed a high correlation between ^probe^*D*_50_ and the retention times (ESI Fig. 6†). The 4′-regioisomer derivatives displayed significantly longer elution times compared to 5′- and 6′-regioisomer based probes (Fig. 2e and ESI Table 6†).

Altogether, our experiments demonstrated that the NGE is present in the 4′-isomer probes and encouraged us to test how it influences the staining performance in living cells.

### Characterization of probe–target interaction *in vitro*

Rhodamine probes are prone to aggregation, which significantly contributes to their fluorogenicity and might impair interaction with the target.4*a* We observed a high correlation between fluorescence increase after binding to tubulin and after SDS addition, which indicates that affinity towards tubulin is sufficient to dissociate the probe from the aggregates (ESI Fig. 7†). In both cases, fluorogenicity changes in the order **TMR** < **580CP** ≈ **610CP** < **SiR**. Next, we carried out a tubulin polymerization assay, which showed that all probes were able to stabilize microtubules and are functional (ESI Fig. 8†).

### Performance of 4′-carboxyrhodamine tubulin probes in living cells

**Fig. 3:**
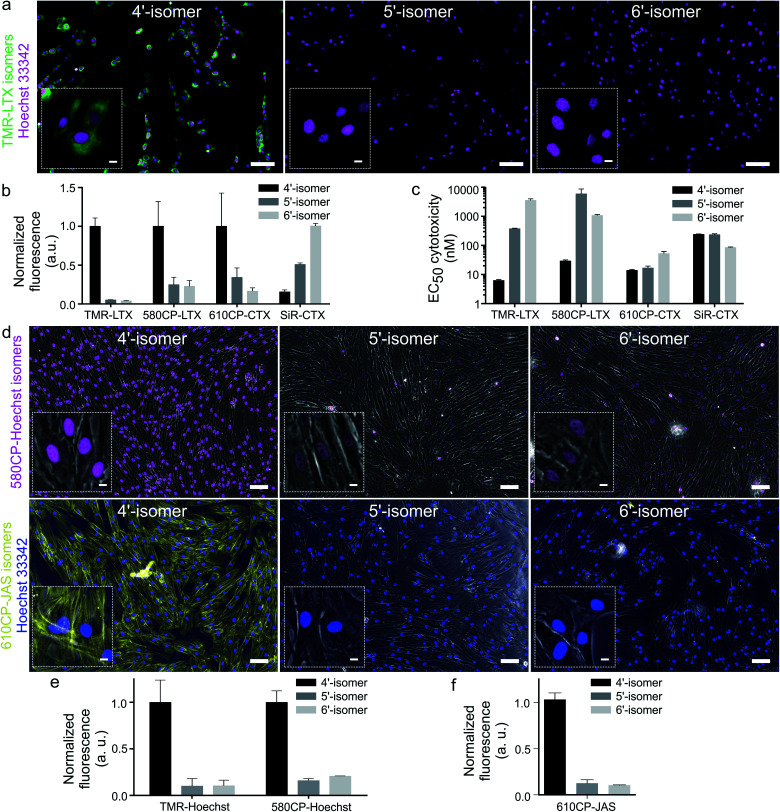
Imaging performance of fluorescent probes based on rhodamine isomers. (a) Wide-field fluorescence microscopy of living primary fibroblasts stained with 100 nM **TMR-LTX** isomers for 1 h at 37 °C. Cells were washed once with HBSS and imaged in DMEM growth media. Inset shows zoomed-in images. Scale bars: 100 μm (large field of view), 10 μm (inset). Hoechst staining is shown in cyan and all tubulin probes are in magenta. (b) Quantification of fluorescence signal in the cytoplasm of living cells stained with tubulin probes. Data are presented as mean ± s.e.m., *N* = 3 independent experiments, each time *n* > 100 cells were quantified. (c) Cytotoxicity of tubulin fluorescent probes presented as half maximal effective concentration (EC_50_) after 24 h incubation at 37 °C in growth media. Cytotoxicity was determined as the fraction of cells containing less than a single set of genetic material (sub G1 DNA content). Data are presented as mean ± s.e.m., *N* = 3 independent experiments, each time *n* > 100 cells were quantified. (d) Wide-field microscopy images of living primary fibroblasts. The cells were stained with 100 nM **4**/**5**/**6-580CP-Hoechst** (magenta) or 100 nM **4**/**5**/**6-610CP-JAS** (yellow) for 1 h at 37 °C, washed once with HBSS and imaged in DMEM media. Insets show zoomed-in images. Scale bars: 100 μm (large field of view), 10 μm (insets). Overlay with phase contrast (grey) images are shown. (e) Quantification of DNA probe fluorescence signal in the nuclei. Data are presented as mean ± s.d., *N* = 3 independent experiments, each *n* > 100 cells. (f) Quantification of **4**/**5**/**6-610CP-JAS** fluorescence signal in the cytoplasm of living cells. Data are presented as mean ± s.d., *N* = 3 independent experiments, each *n* > 100 cells.

Cabazitaxel and Larotaxel are anticancer drugs displaying superior resistance to efflux pumps and brain blood permeability.^17^ The cytotoxicity of tubulin probes is implied by the design, which exploits the attachment of fluorophores to the 3′-position of taxanes.4*a* We hypothesized that NGE enhanced spirolactonization should increase the cell membrane permeability, resulting in stronger staining of tubulin and higher cytotoxicity. Thus, we stained HeLa, U-2 OS and primary human dermal fibroblasts with the series of tubulin probes at 100 nM concentration (Fig. 3a and ESI Fig. 9†). All probes based on 4′-carboxamide dyes, with the exception of **4-SiR-CTX**, stained the cells much more strongly compared to the 5′- or 6′-regioisomer. The improvement of staining ranged from 3- up to 20-fold for primary human fibroblasts (Fig. 3b). In good agreement with these results, all 4′-regioisomer probes, except **4-SiR-CTX**, demonstrated higher cytotoxicity in HeLa cells compared to the 5′- and 6′-regioisomer probes (Fig. 3c, ESI Fig. 10 and Table 7†).

Not only poor membrane permeability, but also active efflux might impede cell staining. To test the effect of multidrug-resistance (MDR) efflux pumps, we took advantage of U-2 OS cells, which are known to express MDR proteins susceptible to a broad specificity inhibitor Verapamil.^18^ Staining with **4-TMR-LTX**, **4-580CP-LTX** and **4-610CP-CTX** was independent of Verapamil, suggesting that these probes are not good substrates of MDR efflux pumps. In contrast, the best performing silicon-rhodamine probe **6-SiR-CTX** was prone to the action of efflux pumps and required Verapamil for efficient staining (ESI Fig. 11†).

### Probes targeting DNA and actin

**Fig. 4:**
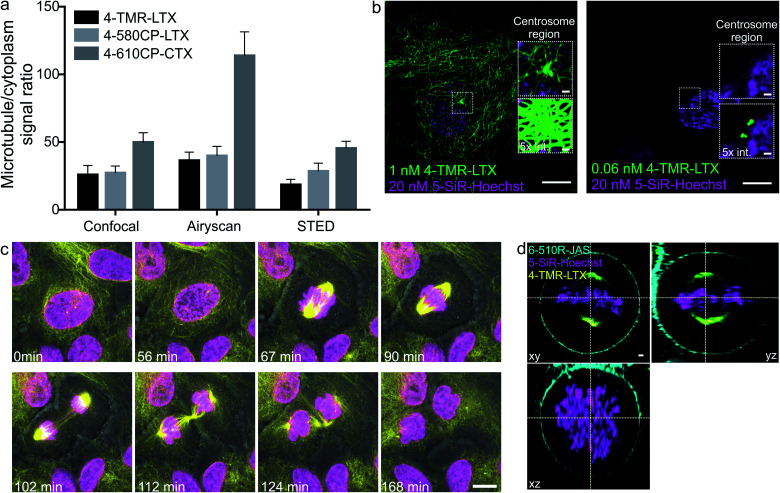
Confocal and Airyscan imaging of living cells stained with rhodamine 4′-isomer probes. (a) Single microtubule and cytoplasm fluorescence signal ratio in living human fibroblasts stained with 100 nM of the indicated probe for 1 h at 37 °C and imaged without probe removal. Data are presented as mean ± s.d., *N* ≥ 3 independent fields of view, each time *n* ≥ 20 microtubules. (b) Zeiss Airyscan images of human primary fibroblasts stained with **5-SiR-Hoechst** and **4-TMR-LTX** for 24 h at 37 °C in growth media at indicated concentrations. Images acquired without probe removal. (c) Cell cycle of human primary fibroblasts stained with 1 nM **4-TMR-LTX** (yellow) and 10 nM **5-610CP-Hoechst** (magenta). Scale bar: 10 μm. Overlay with phase contrast (grey) images are shown. (d) Three-colour ZEISS Airyscan image of living HeLa cells at the metaphase stained with 3 nM **4-TMR-LTX** (yellow), 20 nM **5-SiR-Hoechst** (magenta) and 1000 nM **6-510R-JAS** (cyan). Scale bar: 1 μm.

Superior cell membrane permeability of NGE carrying tubulin probes encouraged us to test the versatility of this approach with the probes targeting DNA and actin. To this end, we synthesized **4-TMR-Hoechst**, **4-580CP-Hoechst** and **4-610CP-JAS**. All these probes stained their targets more intensively compared to 5′- and 6′-regioisomers in living cells (Fig. 4, ESI Fig. 12 and 13†).^19^ DNA probes showed similar affinity to previously reported 5′- and 6′-regioisomers and a single DNA binding mode similar to the 5′-regioisomer (ESI Fig. 12b†).19*b*

Hoechst and its derivatives interfere with DNA synthesis leading to changes in S and G2/M phases.19*b* Jasplakinolide is known to cause multinucleation and apoptosis causing DNA fragmentation.^20^ Knowing these phenotypes, we monitored alterations in the cell cycle progression and identified the cytotoxicity threshold (ESI Fig. 12d and 13c†).

### Confocal and Airyscan microscopy of living cells

Confocal images of tubulin stained with 4′-regioisomers in living cells showed excellent contrast exceeding 50-fold single microtubule to cytoplasm signal ratio even if excess of the probe was not removed (Fig. 4a and ESI Fig. 14†). Airyscan images benefited from increased resolution (∼150 nm) and reached up to 100-fold single microtubule to cytoplasm signal ratio (Fig. 4a and ESI Fig. 15†). This clearly demonstrates excellent permeability, high affinity and selectivity of the taxane probes. A similar improvement was observed for DNA and actin probes (Fig. 4, ESI Fig. 16 and movie 1†).

The **4-TMR-LTX** probe shows a very low cytotoxicity threshold (EC_50_ = 6.3 ± 0.5 nM, Fig. 3c), approaching the cytotoxicity of taxanes, which ranges from 1 to 10 nM^21^ (Table 1). Taxanes are known to accumulate inside the cells up to 1000-fold.^22^ Indeed, we were able to image fibroblasts stained with sub-nanomolar concentrations of **4-TMR-LTX** (Fig. 4b). Despite its relatively low fluorogenicity, a washing step was not required to achieve high contrast. It is worth noting that at picomolar concentrations **4-TMR-LTX** selectively stains the centrosome, likely due to a higher local tubulin concentration in this organelle (Fig. 4b, and ESI movie 2†).

**Table 1:** Properties of the best performing probes

Probe	*λ* abs max (nm)	*λ* em max (nm)	*ε* × 10^3^ (M^–1^ cm^–1^)	QY	*I* STED sat (MW cm^–1^)	Brightness (M^–1^ cm^–1^)	Fl_target_ increase (fold)	Toxicity, EC_50_ (nM)
**Tubulin probes**								
4-TMR-LTX	557	576	65 ± 14	0.56 ± 0.02^*a*^	4.91 ± 0.69	36 100 ± 8100	2.3 ± 0.4	6.3 ± 0.5
4-580CP-LTX	591	612	72 ± 11	0.63 ± 0.01^*a*^	2.29 ± 0.14	45 400 ± 6700	13 ± 3	29 ± 3
4-610CP-CTX	617	638	99 ± 10	0.73 ± 0.02^*a*^	2.75 ± 0.28	72 000 ± 7100	8 ± 1	14 ± 1

**DNA probes**								
4-TMR-Hoechst	558	576	34 ± 3	0.27 ± 0.01^*b*^	—	9100 ± 900	87 ± 7	>4000
4-580CP-Hoechst	589	610	67 ± 4	0.51 ± 0.01^*b*^	—	34 400 ± 1800	34 ± 1	>1000

**Actin probe**								
4-610CP-JAS	618	636	83 ± 4	0.67 ± 0.04^*a*^	—	55 300 ± 2500	40 ± 3	>500

^*a*^Relative quantum yield.

^*b*^Absolute quantum yield.

The spectral separation between **TMR** and **610CP** allows two colour sequential imaging (ESI Fig. 17†). We could resolve all cell cycle stages in 3D while imaging HeLa cells stained with **4-TMR-LTX** and **5-610CP-Hoechst** (Fig. 4c and ESI movie 3†). We exploited **6-510R-JAS**,19a,^23^ **4-TMR-LTX** and **5-SiR-Hoechst** (ref. 19b) for three colour sequential Airyscan Z-stack imaging of mitotic HeLa cells meeting Nyquist criteria (50–50–150 nm in *x*–*y*–*z* axes) without significant bleaching (Fig. 4d and ESI movie 4†).

### Super-resolution STED microscopy of living cells

**Fig. 5:**
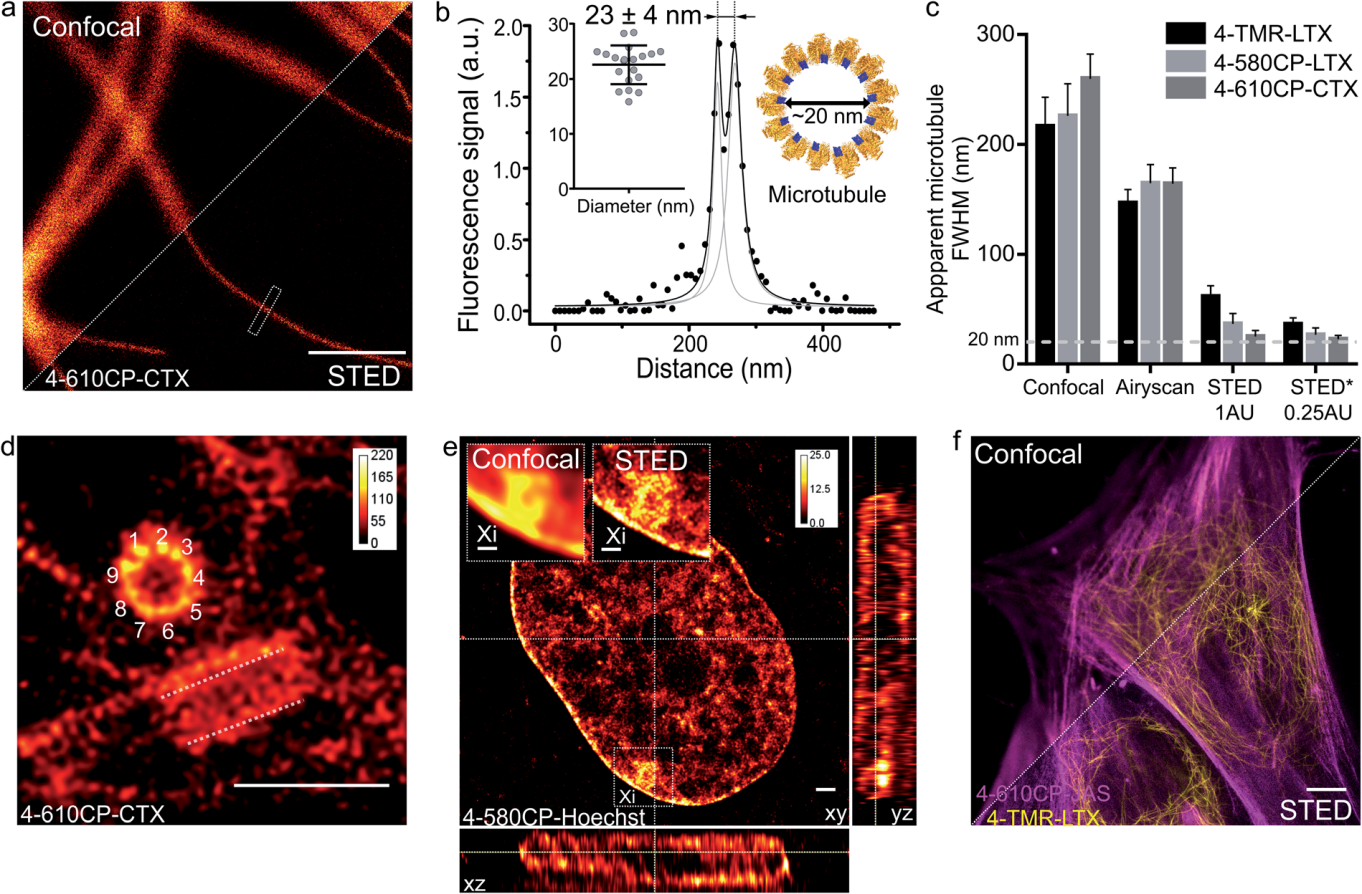
STED nanoscopy imaging of living cells stained with rhodamine 4′-isomer probes. (a) Confocal and STED images of microtubules in human fibroblasts taken with 0.25 AU pinhole. The cells were stained with 100 nM **4-610CP-CTX** for 1 h at 37 °C. Scale bar: 1 μm. (b) Fluorescence intensity profile at the rectangle in panel a. Insets show measured (mean ± s.d., *N* = 20) and predicted diameter from the cryo-electron microscopy model of tubulin (orange) bound to paclitaxel (blue). (c) Apparent microtubule FWHM measured by different microscopy methods. Human fibroblasts stained with 100 nM probes for 1 h at 37 °C. * – diameter measured between two peaks of the fitted intensity profile. Data presented as mean ± s.d., *N* ≥ 3 independent fields of view, each time *n* ≥ 10 microtubules. (d) Nine-fold symmetry of centriole resolved in the deconvolved STED DyMIN^25^ image of U-2 OS cell stained with 1 μM **4-610CP-CTX** for 1 h at 37 °C. White dashed lines mark a second centriole. Scale bar: 1 μm. (e) Deconvolved STED image of human female fibroblast nucleus stained with 100 nM **4-580CP-Hoechst** showing the inactivated X chromosome (Xi). Insets – zoomed-in confocal and STED images of the Xi region. Scale bars: 500 nm (insets), 1 μm (main image). (f) Two-colour STED no-wash image of human fibroblasts stained with 100 nM **4-610CP-JAS** and 10 nM **4-TMR-LTX** for 1 h at 37 °C. Scale bar: 10 μm.

Finally, we applied our new probes in STED nanoscopy. The fluorescence of **TMR** based probes could be inhibited by 660 and 775 nm lasers resulting in significant improvement of the resolution and yielding apparent microtubule diameter below 80 nm in all the tested cases (ESI Fig. 18†). Despite significant fluorescence quenching by the STED laser (10 MW cm^–2^), we could reach up to 45-fold single microtubule to cytoplasm signal ratio while staining with **4-610CP-CTX** (ESI Fig. 19† and 4a). We took advantage of the no-wash conditions and recorded STED images with up to a 100 frames time-course without significant bleaching (ESI movie 5†). Good labelling efficiency and brightness of the probes allowed acquisition of STED images with 0.25 Airy units pinhole at increased resolution (Fig. 5a–c). For the first time we could resolve the microtubule diameter of 23 ± 4 nm in living cells. This value closely matches the dimensions measured with cryo-electron microscopy (Fig. 5b).^24^ In addition, we obtained excellent quality images of tubulin in the centrosome demonstrating the 9-fold symmetry of this organelle (Fig. 5d). 3D STED allowed us to resolve, independent of orientation, the tube-like structure of centrioles stained with **4-610CP-CTX** in living cells without probe removal (ESI Fig. 20 and movie 6†). Using the **4-580CP-Hoechst** probe we visualized chromatin domain clusters of the inactivated X chromosome in primary human fibroblasts (Fig. 5e). Finally, we demonstrate two-colour STED imaging using **4-TMR-LTX** and **4-610CP-JAS** probes (Fig. 5f and ESI Fig. 17d†).

## Conclusions

Due to their excellent photophysical properties and biocompatibility, rhodamines are among the most popular dyes used for synthesis of fluorescent probes for live-cell imaging. Controlling the spirolactonization of rhodamine dyes facilitates cell permeability and increases fluorogenicity of the probes. Herein we exploited the neighbouring group effect (NGE) occurring in the carboxyrhodamines' positional 4′-conjugates for enhancing spirolactonization without any modifications of the dye's core structure. This led to significantly enhanced cell permeability, demonstrating a significant contribution of the fluorophore configuration to the properties of the probe.

Several studies have used ^dye^*D*_50_, the ability to form spirolactones, to guide rational fluorophore design.5b,c,^6^,^26^ In our case this would not have worked; with the exception of **4-SiR-COOH**, ^dye^*D*_50_ values of 4′-, 5′- and 6′-isomers are very close to each other, thus implying no major improvement at the free fluorophore level. In contrast to free dyes, probes are prone to aggregation which can be estimated from fluorescence increase upon addition of a detergent (SDS) that dissolves aggregates (ESI Fig. 4 and 7†). After conjugation to the targeting ligand, one of the neighbouring carboxylic groups is replaced with the carboxamide group, which enhances spirolactonization and considerably increases ^probe^*D*_50_ of 4′-isomers. The spirolactone contributes to high contrast imaging by facilitating the probe's entry into the cell and decreasing the fluorescence of the unbound probe. It is worth noting that the latter mechanism is beneficial but not essential for the high contrast imaging. If a probe can readily cross the cell membrane, it can be efficiently concentrated on the target structure due to the high affinity binding. For example, 1 nM **4-TMR-LTX** is sufficient for efficient tubulin staining (Fig. 4a and b). At such low concentrations, no washing step is required despite the low fluorogenicity of **4-TMR-LTX.** Furthermore, at picomolar concentrations **4-TMR-LTX** selectively stains the centrosome. The unprecedented permeability of the new tubulin probes allowed imaging at nanomolar–picomolar concentrations, which is in sharp contrast to micromolar-high concentrations reported in other studies.5b,c,^6^,^26^,^27^

The high cell permeability of **4-610CP-CTX** allowed exceptionally dense labelling of microtubules and provided sufficiently strong signals for STED imaging with the pinhole set to an extreme value of 0.25 AU. Thereby, to the best our knowledge, for the first time, we resolved microtubule diameters of 23 ± 4 nm in living cells, which matches the value obtained by cryo-EM (Fig. 5a–c).^24^

NGE is beneficial for probes based on more hydrophilic (low ^probe^*D*_50_) **4-TMR-COOH**, **4-580CP-COOH** and **4-610CP-COOH** dyes, but not on a more hydrophobic **4-SiR-COOH**. In **4-SiR-CTX**, NGE pushes ^probe^*D*_50_ outside the water polarity range, causing dominance of a non-absorbing spirolactone, which results in decreased solubility and increased aggregation in water, which competes with the target binding (ESI Table 3†). This manifests in low background fluorescence, but also in weak polymerized tubulin stabilization *in vitro*, low cytotoxicity and poor staining. The delicate balance between spirolactonization–aggregation and target binding is close to ideal for **6-SiR-CTX**, the best performing **SiR**-based probe, which has ^probe^*D*_50_ close to the dielectric constant value of water (ESI Table 3†).

We demonstrated the generality of the NGE cell permeability enhancement by synthesizing probes targeting DNA and actin, which can also operate at low nanomolar concentrations indicating unrestricted permeability, very high affinity and specificity of the new probes. This decreases the chances for unspecific probe accumulation in the membranes. Although invisible, such off-targeting might disturb physiological processes.

In conclusion, we employed the neighbouring group effect in the 4′-regioisomer of rhodamine fluorophores to create, to the best of our knowledge, the most efficient probes for tubulin, actin and DNA labelling in living cells (Table 1 and ESI Table 7†). Their excellent spectroscopic properties, outstanding cell permeability and specificity make them powerful tools for many types of microscopy techniques, including super-resolution methods. This simple approach of positional isomerism is applicable to rhodamine- and fluorescein-type fluorophores, does not require modification of the xanthene ring system and can be used for generating new probes and for improving the performance of existing ones.

## Experimental

### Measurements of absorbance spectra in 1,4-dioxane–water mixtures

Measurements of the absorbance changes in 1,4-dioxane–water mixtures were performed by pipetting 2 μL stock solutions of dyes or probes in DMSO into a 96 glass bottom well plate (11 wells per sample) made from propylene (Corning 3364). To the wells going from right to left 300 μL of 1,4-dioxane–water mixtures containing 100%, 90%, 80%, 70%, 60%, 50%, 40%, 30%, 20%, 10% or 0% 1,4-dioxane was added (if needed, mixtures with 0.3% SDS are used, with an exception in 100% dioxane due to solubility issues). After incubation for 1 hour at room temperature, absorption of solutions in each well was recorded from 320 nm to 850 nm with wavelength step size of 1 nm on a multiwell plate reader Spark® 20 M (Tecan). The background absorption of the glass bottom plate was measured in wells containing only the 1,4-dioxane–water mixture with a similar amount of DMSO and subtracted from the spectra of the samples. *ε*_max_*versus* dielectric constants (*D*) of 1,4-dioxane–water mixtures were plotted.^15^*D*_50_ value was obtained by fitting to dose–response equation EC_50_ as implemented in GraphPad 6.0 software:1
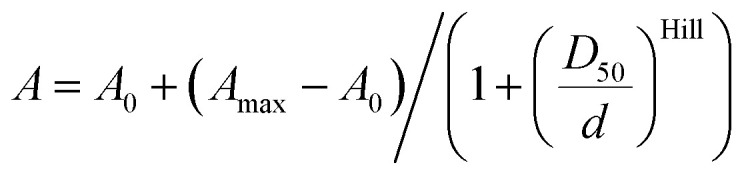
where *A*_0_ – absorbance at *ε*_max_ at *ε*_r_ = 0, *A*_max_ – the highest reached absorbance at *ε*_max_, *d* – dielectric constant of 1,4-dioxane–water mixture at a given point, Hill – Hill slope coefficient determining the steepness of a dose–response curve, *D*_50_ – corresponds to the *d* value that provokes half of the absorbance amplitude (*A*_max_ – *A*_0_).

Data points from the **4-TMR-LTX** titration were fitted to the bell-shaped dose–response curve described by the following equation:2
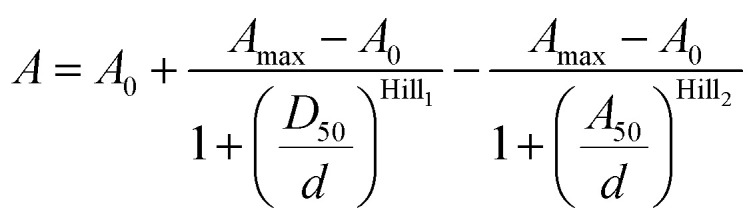
where *A*_0_ – absorbance at *ε*_max_ at *ε*_r_ = 0, *A*_max_ – the highest reached absorbance at *ε*_max_ during the titration experiment. *d* – dielectric constant of 1,4-dioxane–water mixture at a given point, Hill_1_ – Hill slope coefficient determining the steepness of the ascending dose–response curve part, Hill_2_ – Hill slope coefficient determining the steepness of the declining dose–response curve part, *D*_50_ – corresponds to the *d* value that provokes half of the absorbance amplitude (*A*_max_ – *A*_0_) in the ascending dose–response curve part, *A*_50_ – corresponds to the *d* value that provokes half of the absorbance amplitude (*A*_max_ – *A*_0_) in the declining dose–response curve part.

### Quantum chemical calculations

The initial geometries of the studied molecules were generated by using a molecular mechanics method (force field MMF94, steepest descent algorithm) and a systematic conformational analysis as implemented in Avogadro 1.1.1 software. The minimum energy conformer geometries found by molecular mechanics were further optimized with the Gaussian 09 program package^28^ by means of density functional theory (DFT) using the Becke, 3-parameter, Lee–Yang–Parr (B3LYP)^29^ exchange-correlation hybrid functional with 6-311G++(d,p) basis set,^30^ including the polarizable continuum model^31^ to simulate the environment in 1,4-dioxane or water. Furthermore, harmonic vibrational frequencies were calculated to verify the stability of the optimized geometries. All the calculated vibrational frequencies were real (positive) indicating the true minimum of the calculated total potential energy of the optimized system. The computations were performed at the High Performance Computing Center in Göttingen provided by GWDG.

### Maintenance and preparation of cells

Human primary dermal fibroblasts were cultured in high-glucose DMEM (Thermo Fisher, #31053044) and 10% FBS (Thermo Fisher, #10082147) with the presence of 1% penicillin–streptomycin (Sigma, #P0781) in a humidified 5% CO_2_ incubator at 37 °C. HeLa cells were cultured in high-glucose DMEM (Thermo Fisher, #31966047) and 10% FBS (Bio&SELL #S0615), 1 mM sodium pyruvate (Sigma, #S8636) with the presence of 1% penicillin–streptomycin (Sigma, #P0781) in a humidified 5% CO_2_ incubator at 37 °C. The cells were split every 3–4 days or at confluence. Cells were seeded in glass bottom 12-well plates (MatTek, #P12G-1.0-14-F) before imaging experiments. The U2OS cells were cultured in McCoys 5A medium (Thermo fisher #16600082) and 10% FBS (Bio&SELL, #S0615) with the presence of 1 mM sodium pyruvate (Sigma, #S8636) and 1% of penicillin–streptomycin (Sigma #P0781) in a humidified 5% CO_2_ incubator at 37 °C.

Cells were stained with the fluorescent probes in DMEM (Thermo Fisher, #31053044) supplemented with 10% FBS ((Bio&SELL, #S0615)) at 37 °C and 5% CO_2_. If needed, the cells were washed 2 times with HBSS (Lonza, #BE10-527F) and imaged in DMEM with 10% FBS. No-wash experiments were performed in DMEM with 10% FBS after probe addition and incubation for the indicated period of time. For Airyscan experiments, cells were seeded in a 10-well plate (Greiner bio-one, #543079) 1 day before staining. The probes were applied to the cells in DMEM medium, and after 1 h or overnight incubation cells were imaged without washing.

### STED microscope with a 775 nm laser

Comparative confocal and STED images were acquired on an Abberior STED 775 QUAD scanning microscope (Abberior Instruments GmbH) equipped with 561 nm and 640 nm 40 MHz pulsed excitation lasers, a pulsed 775 nm 40 MHz STED laser, and an UPlanSApo 100×/1.40 Oil objective. The following detection windows were used: for the TMR/580CP channel 615/20 nm, and for the 610CP/SiR channel 685/70 nm. In this setup, voxel size was 15–30 nm in the *xy* plane and 150 nm in the *z*-axis for 2D STED images. For the STED imaging with a 0.25 AU pinhole, the pixel size was set to 7 × 7 nm and images were acquired with 2 line accumulation. Laser powers were optimized for each sample. 3D STED images were acquired using a pinhole set to 0.8 AU, voxel size set to 40 × 40 × 40 nm, 3D STED doughnut set to 90%, with single line accumulation and *xzy* scanning mode.

Estimation of the STED effect was performed by varying the STED laser power from 0 to 100% while measuring cells stained with tubulin probes. Obtained data were fitted using GraphPad Prism 6 to the following equation:3
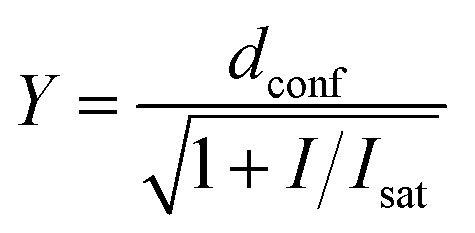
where *d*_conf_ – confocal resolution, *I* – STED laser intensity power, *I*_sat_ – saturating STED laser intensity power.

### STED microscope with a 660 nm laser

STED images with a 660 nm STED laser were acquired on a Leica TCS SP8 STED 3X scanning microscope (Leica Microsystems) equipped with an HC PL APO CS2 100×/1.40 oil objective and the incubator set to 37 °C and 5% CO_2_. The **4-TMR-LTX** probe was excited with the 561 nm, 80 MHz pulsed line of a white light laser, de-excited with a continuous wave 660 nm STED laser and detected in the 600/50 nm window. In this setup, the pixel size was set to 23 nm in the xy plane. The STED effect was estimated as described above.

### Airyscan microscope

Live-cell imaging was performed on a Zeiss LSM880 system (Carl Zeiss) equipped with an oil immersion Plan-Apochromat 63×/1.40 Oil Corr M27 objective and the incubator set to 37 °C and 5% CO_2_. The following excitation lasers in combination with confocal or Airyscan acquisition regimes were used: argon laser 488 nm for 510R probes, 561 nm diode laser for TMR and 580CP probes, and 633 nm diode laser for 610CP and SiR probes. For TMR and 610CP, a combination of BP 570–620 + LP 645 filters with dichroic beam splitter SBS SP 615 in the TMR channel was used. The TMR, 510R and SiR combination was excited and scanned in sequence using BP 495–550 + LP 570 filters. Laser powers were optimized for each sample. Detailed excitation and detection schemes are shown in ESI Fig. 16.†

### Processing and visualization of acquired images

All acquired or reconstructed images were processed and visualized using Fiji.^32^ Line profiles were measured using the “straight line” tool with the line width set to 3 pixels.

For the signal measurements, image files were converted to a TIF file using Fiji and analyzed with CellProfiler 3.1.8 (ref. 33), where the pipeline identified the nuclear region and measured the mean signal in this region. Background signal was measured in the region which is 3 pixels (450 nm) away from the nuclear border and 7 pixels (1050 nm) wide. The background subtracted signal was processed with GraphPad Prism 6.

Actin/tubulin cytosolic signal was estimated using CellProfiler v.3.1.8. Briefly, the probe channel was smoothed using a median filter, nuclei were identified in the DAPI channel and were used as seeds to find cell outlines in a smoothed actin channel. Background is defined as a lower quartile of pixel intensity in the area not covered by cells in the original probe channel. The background was subtracted from the original probe channel, and actin/tubulin staining was measured as mean pixel intensity per object in the background-corrected probe channel. Statistical analysis was performed using GraphPad Prism 6.

## Conflicts of interest

GL has filed a patent application on SiR derivatives. JB, GK, RG and GL have filed a patent application on 4′-isomers.

## Supplementary Material

Supplementary information

Supplementary movie

Supplementary movie

Supplementary movie

Supplementary movie

Supplementary movie

Supplementary movie
